# Linear models enable powerful differential activity analysis in massively parallel reporter assays

**DOI:** 10.1186/s12864-019-5556-x

**Published:** 2019-03-12

**Authors:** Leslie Myint, Dimitrios G. Avramopoulos, Loyal A. Goff, Kasper D. Hansen

**Affiliations:** 1 Department of Biostatistics, Johns Hopkins Bloomberg School of Public Health, 615 N. Wolfe St, E3527, Baltimore, MD 21212 USA; 2 McKusick-Nathans Institute of Genetic Medicine, Johns Hopkins School of Medicine, Baltimore, USA; 3 Department of Neuroscience, Johns Hopkins School of Medicine, Baltimore, USA; 4 Department of Mathematics, Statistics, and Computer Science, Macalester College, 1600 Grand Ave, Saint Paul, MN 55105 USA

**Keywords:** Massively parallel reporter assays, Statistics, Enhancer

## Abstract

**Background:**

Massively parallel reporter assays (MPRAs) have emerged as a popular means for understanding noncoding variation in a variety of conditions. While a large number of experiments have been described in the literature, analysis typically uses ad-hoc methods. There has been little attention to comparing performance of methods across datasets.

**Results:**

We present the mpralm method which we show is calibrated and powerful, by analyzing its performance on multiple MPRA datasets. We show that it outperforms existing statistical methods for analysis of this data type, in the first comprehensive evaluation of statistical methods on several datasets. We investigate theoretical and real-data properties of barcode summarization methods and show an unappreciated impact of summarization method for some datasets. Finally, we use our model to conduct a power analysis for this assay and show substantial improvements in power by performing up to 6 replicates per condition, whereas sequencing depth has smaller impact; we recommend to always use at least 4 replicates. An R package is available from the Bioconductor project.

**Conclusions:**

Together, these results inform recommendations for differential analysis, general group comparisons, and power analysis and will help improve design and analysis of MPRA experiments.

## Background

**Fig. 1:**
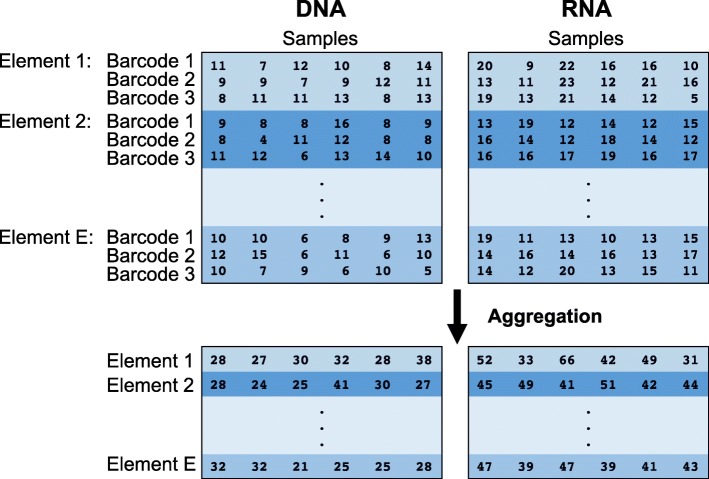
Structure of MPRA data. Thousands of putative regulatory elements can be assayed at a time in an MPRA experiment. Each element is linked to multiple barcodes. A plasmid library containing these barcoded elements is transfected into several cell populations (samples). Cellular DNA and RNA can be isolated and sequenced. The barcodes associated with each putative regulatory element can be counted to obtain relative abundances of each element in DNA and RNA. The process of aggregation sums counts over barcodes for element in each sample. Aggregation is one method for summarizing barcode level data into element level data

Noncoding regions in the human genome represent the overwhelming majority of genomic sequence, but their function remains largely uncharacterized. Better understanding of the functional consequences of these regions has the potential to greatly enrich our understanding of biology. It is well understood that some noncoding regions are regulatory in nature. It has been straightforward to experimentally test the regulatory ability of a given DNA sequence with standard reporter assays, but these assays are low throughout and do not scale to the testing of large numbers of sequences. Massively parallel reporter assays (MPRAs) have emerged as a high-throughput means of measuring the ability of sequences to drive expression [1, 2]. These assays build on the traditional reporter assay framework by coupling each putative regulatory sequence with several short DNA tags, or barcodes, that are incorporated into the RNA output. These tags are counted in the RNA reads and the input DNA, and the resulting counts are used to quantify the activity of a given putative regulatory sequence, typically involving the ratio of RNA counts to DNA counts (Fig. 1). The applications of MPRA have been diverse, and there have been correspondingly diverse and ad hoc methods used in statistical analysis.

There are three broad categories of MPRA applications: (1) characterization studies, (2) saturation mutagenesis, and (3) differential analysis. (1) Characterization studies examine thousands of different putative regulatory elements that have a wide variety of sequence features and try to correlate these sequence features with measured activity levels [3–10]. Typical statistical analyses use regression to study the impact of multiple features simultaneously. They also compare continuous activity measures or categorized (high/low) activity measures across groups using paired and unpaired t-, rank, Fisher’s exact, and chi-squared tests. (2) Saturation mutagenesis studies look at only a few established enhancers and examine the impact on activity of every possible mutation at each base as well as interactions between these mutations [11–17]. Analyses have uniformly used linear regression where each position in the enhancer sequence is a predictor. (3) Differential analysis studies look at thousands of different elements, each of which has two or more versions. Versions can correspond to allelic versions of a sequence [18–20] or different environmental contexts [21], such as different cell or tissue types [22]. These studies have compared different sequence versions using paired t-tests, rank sum tests, and Fisher’s exact test (FET) (by pooling counts over biological replicates).

Despite the increasing popularity of this assay, guiding principles for statistical analysis have not been put forth. Researchers use a large variety of ad hoc methods for analysis. For example, there has been considerable diversity in the earlier stages of summarization of information over barcodes. Barcodes are viewed as technical replicates of the regulatory element sequences, and groups have considered numerous methods for summarizing barcode-level information into one activity measure per enhancer. On top of this, a large variety of statistical tests are used to make comparisons.

Recently, a method called QuASAR-MPRA was developed to identify regulatory sequences that have allele-specific activity [23]. This method uses a beta-binomial model to model RNA counts as a function of DNA counts, and it provides a means for identifying sequences that show a significant difference in regulatory activity between two alleles. While it provides a framework for two group differential analysis within MPRAs, QuASAR-MPRA is limited in this regard because experiments might have several conditions and involve arbitrary comparisons.

To our knowledge, no method has been developed that provides tools for general purpose differential analysis of activity measures from MPRA. General purpose methods are ones that can flexibly analyze data from a range of study designs. We present mpralm, a method for testing for differential activity in MPRA experiments. Our method uses linear models as opposed to count-based models to identify differential activity. This approach provides desired analytic flexibility for more complicated experimental designs that necessitate more complex models. It also builds on an established method that has a solid theoretical and computational framework [24]. We show that mpralm can be applied to a wide variety of MPRA datasets and has good statistical properties related to type I error control and power. Furthermore, we examine proper techniques for combining information over barcodes and provide guidelines for choosing sample sizes and sequencing depth when considering power. Our method is open source and freely available in the *mpra* package for R on the Bioconductor repository [25].

## Results

### The structure of MPRA data and experiments

MPRA data consists of measuring the activity of some putative regulatory sequences, henceforth referred to as “elements”. First a plasmid library of oligos is constructed, where each element is coupled with a number of short DNA tags, or barcodes. This plasmid library is then transfected into one or more cellular contexts, either as free-floating plasmids or integrated into the genome [21]. Next, RNA output is measured using RNA sequencing, and DNA output as a proxy for element copy number is measured using DNA sequencing (occasionally, element copy number is unmeasured), giving the data structure shown in Fig. 1. The log-ratio of RNA to DNA counts is commonly used as an activity outcome measure.

Since each element is measured across a number of barcodes, it is useful to summarize this data into a single activity measure *a* for a single element in a single sample. Multiple approaches have been proposed for this summarization step. We consider two approaches. First is averaging, where a log-ratio is computed for each barcode, then averaged across barcodes. This treats the different barcodes as technical replicates. The second approach is aggregation, where RNA and DNA counts are each summed across barcodes, followed by formation of a log-ratio. This approach effectively uses the barcodes to simply increase the sequencing counts for that element.

In our investigation of the characteristics of MPRA data we use a number of datasets listed in Table 1. We have divided them into 3 categories. Two categories are focused on differential analysis: one on comparing different alleles and one on comparing the same element in different conditions (retina vs. cortex and episomal vs. chromosomal integration). The two allelic studies naturally involve paired comparisons in that the two elements being compared are always measured together in a single sample (which is replicated). We also use two saturation mutagenesis experiments.

**Table 1:** Datasets

Dataset	Description	Cell culture	Replicates	Barcodes
Differential analysis: alleles				
Tewhey	Study of 39,479 oligos coming from 29,173 variants	NA12878 (LCL)	NA12878: 5	79k pool: ∼73
	to follow up on prior eQTL results.	NA19239 (LCL)	NA19239: 3	7.5k pool: ∼350
	Large initial oligo pool: 79k. Second pool: 7.5k.	HepG2	HepG2: 5	
Ulirsch	Study of 2756 variants in strong LD with 75 main	K562, K562 with	K562: 6	14
	variants to identify loci that affect RBC traits.	GATA1 over-expr.	K562+GATA1: 4	
Differential analysis: conditions				
Inoue	Comparison of episomal and lentiviral MPRA.	HepG2	3	Max: 99
Shen	Study of tissue specificity of cis-regulatory	Mouse retina and	3	∼8
	element in-vivo in mouse.	cerebral cortex		
Saturation mutagenesis				
Melnikov	Two inducible enhancers:	HEK293T	Single: 2	Single: 13
	(1) a synthetic cAMP-regulated enhancer and		Multi: 2	Multi: 1
	(2) the virus-inducible interferon-beta enhancer.			
	Single-hit scanning alters one base at a time.			
	Multi-hit sampling alters several bases at a time.			
Kheradpour	Study of 2104 wild-type sequences and 3314 variant	K562	2	10
	sequences containing targeted motif disruptions to	HepG2		
	understand base-level effects in motifs.			

All datasets are publicly available, see the relevant publication

### The variability of MPRA data depends on element copy number

It is well established that count data from RNA sequencing studies exhibit a mean-variance relationship [26]. On the log scale, low counts are more variable across replicates than high counts, at least partly due to inherent Poisson variation in the sequencing process [27, 28]. This relationship has been leveraged in both count-based analysis methods [29, 30] and, more recently, linear model-based methods [24]. For count-based methods, this mean-variance relationship helps improve dispersion estimates, and for linear model-based methods, the relationship allows for estimation of weights reflecting inherent differences in variability for count observations in different samples and genes.

**Fig. 2:**
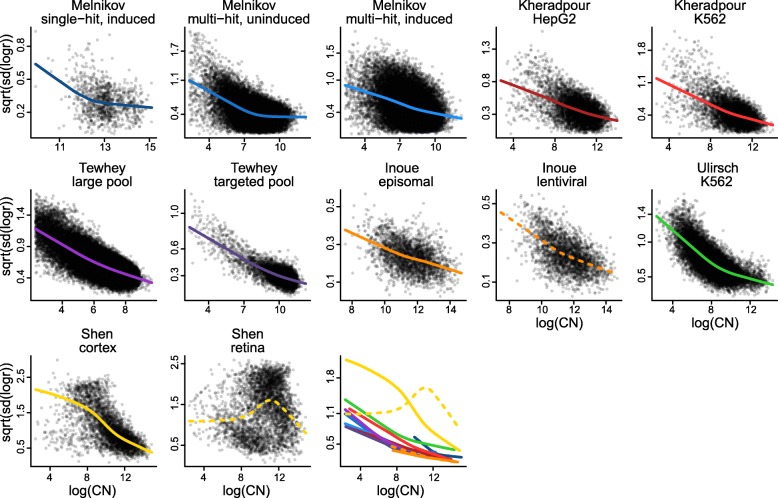
Variability of MPRA activity measures depends on element copy number. For multiple publicly available datasets we compute activity measures of putative regulatory element as the log2 ratio of aggregated RNA counts over aggregated DNA counts. Each panel shows the relationship between variability (across samples) of these activity measures and the average log2 DNA levels (across samples). Smoothed relationships are lowess curves representing the local average variability. The last plot shows all lowess curves on the same figure

Because MPRAs are fundamentally sequencing assays, it is useful to know whether similar variance relationships hold in these experiments. Due to the construction of MPRA libraries, each element is present in a different (random) copy number, and this copy number ought to impact both background and signal measurements from the element. We are therefore specifically interested in the functional relationship between element copy number and the variability of the activity outcome measure. As outcome measure we use the log-ratio of RNA counts to DNA counts (aggregate estimator), and we use aggregated DNA counts, averaged across samples, as an estimate of DNA copy number. We compute empirical standard deviations of the library size-corrected outcome measure across samples. In Fig. 2 we depict this relationship across the previously discussed publicly available datasets (Table 1). For all datasets, with one exception, there is higher variation associated with lower copy number. The functional form is reminiscent of the mean-variance relationship in RNA sequencing data [24], despite that we here show variance of a log-ratio of sequencing counts.

### Statistical modeling of MPRA data

To model MPRA data we propose to use a simple variant of the voom methodology [24], proposed for analysis of RNA sequencing data. This methodology is based on standard linear models, which are coupled with inverse variance weights representing the mean-variance relationship inherent in RNA sequencing data. The weights are derived from smoothing an empirical mean-variance plot. Similar to voom, we propose to use linear models to model log-ratio activity data from MPRAs, but we estimate weights by smoothing the relationship between empirical variance of the log-ratios and log-DNA copy number, as depicted in Fig. 2. We call this approach mpralm.

The current literature on analysis of MPRA experiments contains many variant methods (see Introduction). To evaluate mpralm, we compare the method to the following variants used in the literature: QuASAR-MPRA, t-tests, and Fisher’s exact test (FET). QuASAR-MPRA is a recently developed method that is targeted for the differential analysis of MPRA data [23]. It specifically addresses a two group differential analysis where the two groups are elements with two alleles and uses base-calling error rate in the model formulation. It collapses count information across samples to create three pieces of information for each element: one count for RNA reads for the reference allele, one count for RNA reads for the alternate allele, and one proportion that gives the fraction of DNA reads corresponding to the reference allele. Fisher’s exact test similarly collapses count information across samples. To test for differential activity, a 2-by-G table is formed with RNA and DNA designation forming one dimension and condition designation (with *G* groups) in the second dimension. The t-test operates on the log-ratio outcomes directly; we use the aggregate estimator to summarize over barcodes. Either a paired or unpaired t-test is used based on experimental design.

Both edgeR and DESeq2 are popular methods for analysis of RNA-sequencing data represented as counts. The two methods are both built on negative binomial models, and both attempt to borrow information across genes. These methods allow for the inclusion of an offset. Because both methods use a logarithmic link function, including log-DNA as an offset allows for the modeling of log-ratios of RNA to DNA. This makes these methods readily applicable to the analysis of MPRA data, and they carry many of the same advantages as mpralm. In addition to QuASAR, t-tests, and Fisher’s exact test, we examine the performance of edgeR and DESeq2 for differential activity analysis in our evaluations. We point out that although our application of edgeR and DESeq2 to MPRA data is straightforward, it has not been used in this way so far in the literature. Tewhey et al. [19] uses DESeq2 to perform differential expression analysis of RNA counts relative to DNA counts within a single condition. This assesses whether the regulatory elements have activating or repressive activity, but it does not assess whether the activity of regulatory elements differs between conditions. We remind the community of the ability to use offsets in the edgeR and DESeq2 negative binomial models, and explore a new use of these models for MPRA data.

### mpralm is a powerful method for differential analysis

First, we focus on evaluating the performance of mpralm for differential analysis. We compare to QuASAR-MPRA, t-tests, Fisher’s exact test, edgeR, and DESeq2. We use four of the previously discussed studies, specifically the Tewhey, Inoue, Ulirsch, and Shen studies. Two of these studies (Tewhey, Ulirsch) focus on comparing the activity of elements with two alleles, whereas the other two (Inoue, Shen) compare the activity of each element in two different conditions. For the allelic studies, we use a random effects model for mpralm and paired t-tests. In the random effects model, we estimate the correlation between the multiple alleles for a given single nucleotide polymorphism (SNP). This correlation estimate improves the estimation of element-wise variances used in testing for differences between conditions. Both Tewhey et al. [19] and Ulirsch et al. [18] compare alleles in different cellular contexts; we observe similar behavior of all evaluations in all contexts (data not shown) and have therefore chosen to depict results from one cellular context for both of these studies. For the Tewhey dataset we depict results both from a large pool of elements used for initial screening and a smaller, targeted pool.

**Fig. 3:**
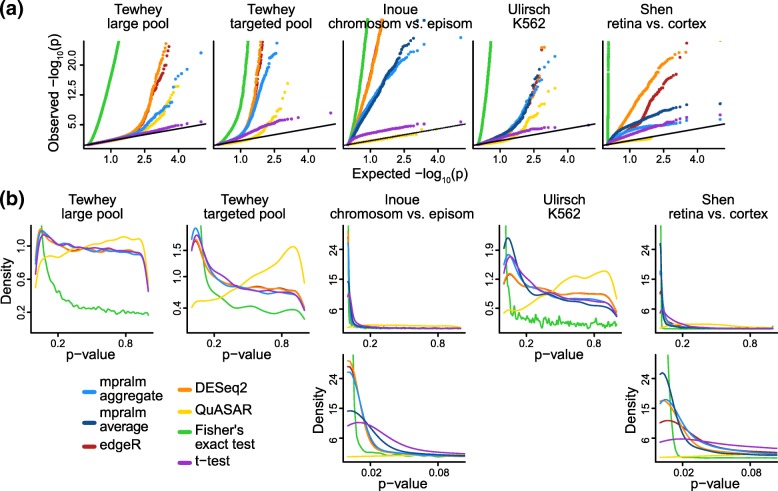
Comparison of detection rates and *p*-value calibration over datasets. (**a**) QQ-plots (row 1), and (**b**) density plots (rows 2 and 3) for *p*-values for all datasets, including a zoom of the [0,0.1] interval for some datasets (row 3). Over all datasets, most methods show *p*-values that closely follow the classic mixture of uniformly distributed *p*-values with an enrichment of low *p*-values for differential elements. For the datasets which had barcode level counts (Inoue, Ulirsch, and Shen), we used two types of estimators of the activity measure (log-ratio of RNA/DNA) with mpralm, shown in light and dark blue

Figure 3 shows *p*-value distributions that result from running all methods on the 5 real datasets. Across these datasets, all methods except for QuASAR show a well-behaved *p*-value distribution; high *p*-values appear uniformly distributed, and there is a peak at low *p*-values. QuASAR-MPRA consistently shows conservative *p*-value distributions. This feature of the *p*-value distributions is not apparent from the QQ-plots (Fig. 3, first row) that the authors use to evaluate their method [23]. Fisher’s exact test has a very high peak around zero, likely due to the extreme sensitivity of the test with high counts. We examine mpralm using both an average estimator and an aggregation estimator for summarizing across barcodes; this cannot be done for the Tewhey dataset where we do not have access to barcode-level data. To fully interpret these *p*-value distributions, we need to assess error rates. For example, the large number of rejections achieved using Fisher’s exact test may be associated with a large number of errors.

**Fig. 4:**
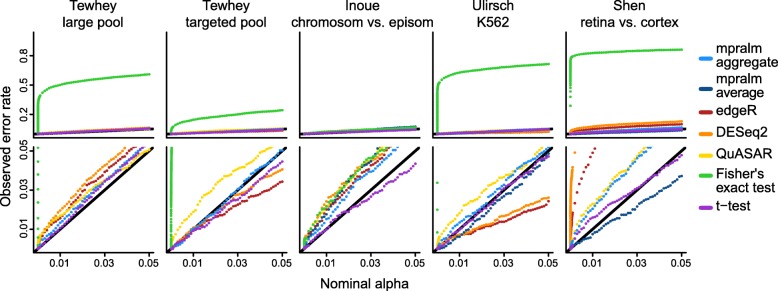
Empirical type I error rates. Type I error rates were estimated for all methods with simulated null data (Methods). For the datasets which had barcode level counts (Inoue, Ulirsch, and Shen), we used two types of estimators of the activity measure (aggregate and average estimator) with mpralm, shown in dark and light blue

To estimate empirical dataset-specific type I error rates, we simulated count data that gave rise to null comparisons for each regulatory element (Methods). With all comparisons being null, we estimate the dataset-dependent type I error rate for each method as the fraction of rejected null hypotheses at a given nominal level. Figure 4 shows these estimated error rates (based on simulated data). We observe that Fisher’s exact test has wildly inflated type I error, presumably because MPRA count data are overdispersed and because exact tests for large count data can be very sensitive. The other methods are much closer to being calibrated, although there is some variability from dataset to dataset. Generally, QuASAR-MPRA seems to be slightly liberal across datasets. The t-test is close to consistently calibrated. DESeq2 and edgeR are the most variable in their calibration; in particular, they are fairly conservative for the Ulirsch dataset and liberal for the Shen dataset. mpralm falls between the t-test and edgeR/DESeq2 for calibration. For high throughput assays, there is also interest in type I error rate calibration at very low nominal levels (e.g. Bonferroni-adjusted levels). In Table 2, we show estimated error rates for very low nominal error rates (roughly corresponding to *α*=0.05/3000,0.05/5000,0.05/7000,0.05/9000). We see that at these stringent levels typical of multiple testing situations in these assays, mpralm with the aggregate estimator and the t-test tend to not make any errors.

**Table 2:** Observed type I error rates for low nominal error rates

Nominal	edgeR	DESeq2	QuASAR	mpralm	mpralm	FET	t-test
error rate				(agg)	(avg)		
Tewhey - large							
1.62e-05	8.21e-04	8.22e-04	1.97e-03	1.64e-04	NA	2.82e-01	0
	(5/6088)	(5/6084)	(12/6088)	(1/6088)		(1718/6088)	(0/6088)
1.03e-05	6.57e-04	8.22e-04	1.64e-03	0	NA	2.73e-01	0
	(4/6088)	(5/6084)	(10/6088)	(0/6088)		(1663/6088)	(0/6088)
7.25e-06	4.93e-04	8.22e-04	1.64e-03	0	NA	2.66e-01	0
	(3/6088)	(5/6084)	(10/6088)	(0/6088)		(1618/6088)	(0/6088)
6.25e-06	4.93e-04	6.57e-04	1.64e-03	0	NA	2.62e-01	0
	(3/6088)	(4/6084)	(10/6088)	(0/6088)		(1597/6088)	(0/6088)
Tewhey - targeted							
1.62e-05	5.71e-04	5.71e-04	1.71e-03	5.71e-04	NA	2.57e-02	0
	(2/3503)	(2/3503)	(6/3503)	(2/3501)		(90/3503)	(0/3503)
1.03e-05	5.71e-04	5.71e-04	1.71e-03	5.71e-04	NA	2.37e-02	0
	(2/3503)	(2/3503)	(6/3503)	(2/3501)		(83/3503)	(0/3503)
7.25e-06	5.71e-04	5.71e-04	1.43e-03	5.71e-04	NA	2.14e-02	0
	(2/3503)	(2/3503)	(5/3503)	(2/3501)		(75/3503)	(0/3503)
6.25e-06	5.71e-04	5.71e-04	1.43e-03	5.71e-04	NA	2.00e-02	0
	(2/3503)	(2/3503)	(5/3503)	(2/3501)		(70/3503)	(0/3503)
Inoue							
1.62e-05	1.64e-03	2.05e-03	4.1e-04	0	4.1e-04	3.69e-03	4.1e-04
	(4/2440)	(5/2440)	(1/2440)	(0/2440)	(1/2440)	(9/2440)	(1/2440)
1.03e-05	1.64e-03	2.05e-03	4.1e-04	0	4.1e-04	3.28e-03	4.1e-04
	(4/2440)	(5/2440)	(1/2440)	(0/2440)	(1/2440)	(8/2440)	(1/2440)
7.25e-06	8.20e-04	2.05e-03	4.1e-04	0	4.1e-04	2.87e-03	4.1e-04
	(2/2440)	(5/2440)	(1/2440)	(0/2440)	(1/2440)	(7/2440)	(1/2440)
6.25e-06	8.20e-04	2.05e-03	4.1e-04	0	4.1e-04	2.46e-03	4.1e-04
	(2/2440)	(5/2440)	(1/2440)	(0/2440)	(1/2440)	(6/2440)	(1/2440)
Ulirsch							
1.62e-05	0	0	1.09e-03	0	0	4.14e-01	0
	(0/2756)	(0/2756)	(3/2756)	(0/2756)	(0/2756)	(1140/2756)	(0/2756)
1.03e-05	0	0	7.26e-04	0	0	4.06e-01	0
	(0/2756)	(0/2756)	(2/2756)	(0/2756)	(0/2756)	(1119/2756)	(0/2756)
7.25e-06	0	0	7.26e-04	0	0	3.96e-01	0
	(0/2756)	(0/2756)	(2/2756)	(0/2756)	(0/2756)	(1092/2756)	(0/2756)
6.25e-06	0	0	7.26e-04	0	0	3.93e-01	0
	(0/2756)	(0/2756)	(2/2756)	(0/2756)	(0/2756)	(1083/2756)	(0/2756)
Shen							
1.62e-05	2.45e-03	1.67e-02	2.98e-04	0	3e-04	7.39e-01	0
	(8/3264)	(51/3052)	(1/3358)	(0/3286)	(1/3337)	(2412/3264)	(0/3264)
1.03e-05	2.45e-03	1.54e-02	0	0	3e-04	7.33e-01	0
	(8/3264)	(47/3052)	(0/3358)	(0/3286)	(1/3337)	(2393/3264)	(0/3264)
7.25e-06	2.14e-03	1.31e-02	0	0	3e-04	7.30e-01	0
	(7/3264)	(40/3052)	(0/3358)	(0/3286)	(1/3337)	(2384/3264)	(0/3264)
6.25e-06	1.84e-03	1.21e-02	0	0e+00	0e+00	7.29e-01	0
	(6/3264)	(37/3052)	(0/3358)	(0/3286)	(0/3337)	(2379/3264)	(0/3264)

To investigate the trade-off between observed power (number of rejected tests) and type I error rates, we combine these quantities in two ways: (1) we look at the number of rejections as a function of observed type I error rates and (2) we look at estimated FDR as a function of the number of rejections. Specifically, we use the error rates we estimate using the simulation (Fig. 4) to adjust the comparison of the observed number of rejections (Fig. 3).

**Fig. 5:**
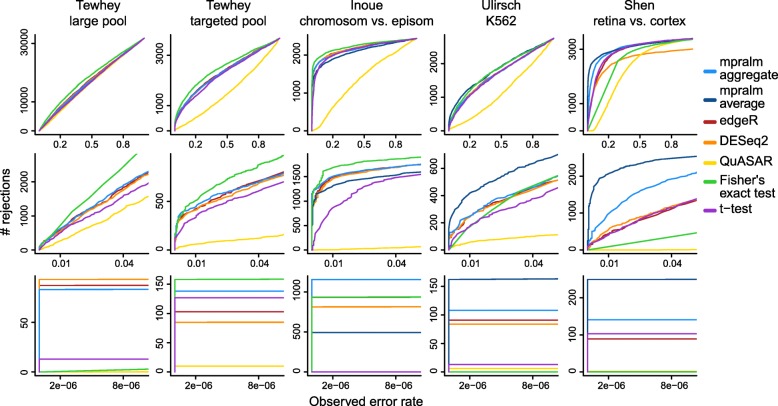
Number of rejections as a function of observed error rate. To compare the observed detection (rejection) rates of the methods fairly, we compare them at the same observed type I error rates, estimated in Fig. 4. The bottom two rows are zoomed-in versions of the top row. We see that mpralm, edgeR, and DESeq2 consistently have the highest detection rates

In Fig. 5 we display the number of rejections as a function of calibrated (using simulation, Fig. 4) type I error rates. For a fixed calibrated error rate, we interpret a high number of rejections to suggest high power (since the error rates are calibrated to be the same). QuASAR-MPRA shows poor performance on this metric across datasets because it generates such conservative *p*-values. FET shows good performance for the Tewhey and Inoue datasets, but this is caused by its extremely liberal *p*-value distribution. It performs more poorly relative to the other methods in the Ulirsch and Shen datasets. Across these datasets, mpralm tends to have the best performance, particularly at low nominal error rates (Fig. 4, rows 2 and 3). However, edgeR and DESeq2 are close behind.

**Fig. 6:**
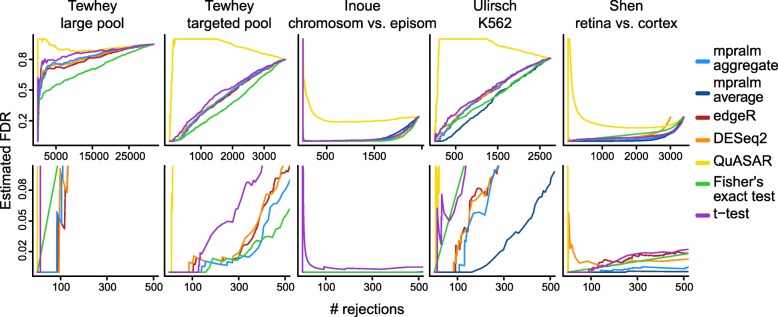
Estimated FDR. For each dataset and method, the false discovery rate is estimated as a function of the number of rejections. This requires estimation of the proportion of true null hypotheses (Methods). The bottom row is a zoomed-in version of the top row

If we know the proportion of true null hypotheses, *π*_0_, and the true type I error rate, we can compute false discovery rates (FDR). The true *π*_0_ is an unknown quantity, but we estimate it using a method developed by Phipson et al. [31]. The true type I error rate is also an unknown quantity, but we estimated it via a realistic simulation as described earlier (Fig. 4). Using these estimates, we compute an estimated FDR (Methods). In Fig. 6 the estimated FDR (for a given *π*_0_) is displayed as a function of the number of rejections. QuASAR-MPRA, t-tests, and Fisher’s exact test tend to have the highest false discovery rates. mpralm tends to have the lowest FDRs, with edgeR and DESeq2 close behind. For the Inoue dataset, all methods have very low FDR, presumably because a very high fraction of elements are expected to be differential given the extreme expected differences between the comparison groups.

In conclusion, we observe that Fisher’s exact test has too high of an error rate and that QuASAR-MPRA is underpowered; based on these results we cannot recommend either method. T-tests perform better than these two methods but are still outperformed by mpralm, edgeR, and DESeq2. These methods have similar performance, but mpralm seems to have slightly better performance than the latter two in terms of consistency of type I error calibration and power.

### Comparison of element rankings between methods

While power and error calibration are important evaluation metrics for a differential analysis method, they do not have a direct relation with element rankings, which is often of practical importance. for the top performing methods in our evaluations (mpralm, t-test, edgeR and DESeq2) we examine rankings in more detail.

**Fig. 7:**
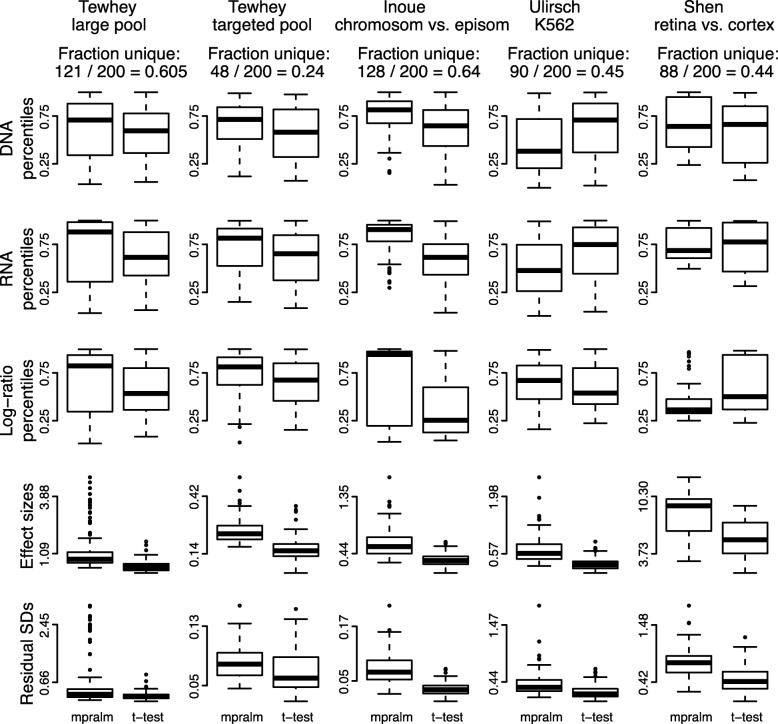
Distribution of quantities related to statistical inference in top ranked elements with mpralm and t-test. MPRA elements that appear in the top 200 elements with one method but not the other are examined here. For these uniquely top ranking elements, the DNA, RNA, and log-ratio percentiles are shown in the first three rows. The effect sizes (difference in mean log-ratios) and residual standard deviations are shown in the last two rows. Overall, uniquely top ranking elements for the t-test tend to have lower log-ratio activity measures, effect sizes, and residual standard deviations

We observe fairly different rankings between mpralm and the t-test and examine drivers of these differences in Fig. 7. For each dataset, we find the MPRA elements that appear in the top 200 elements with one method but not the other. We will call these uniquely top ranking elements, and they make up 24% to 64% of the top 200 depending on dataset. For most datasets, DNA, RNA, and log-ratio activity measures are higher in uniquely top ranking mpralm elements (top three rows of Fig. 7). It is desirable for top ranking elements to have higher values for all three quantities because higher DNA levels increase confidence in the activity measure estimation, and higher RNA and log-ratio values give a stronger indication that a particular MPRA element has regulatory activity. In the last two rows of Fig. 7, we compare effect sizes and variability measures (residual standard deviations). The t-test uniformly shows lower variability but also lower effect sizes for its uniquely top ranking elements. This follows experience from gene-expression studies where standard t-tests tend to underestimate the variance and thereby exhibit t-statistics which are too large, leading to false positives. In MPRA studies, as with most other high-throughput studies, it is typically more useful to have elements with high effect sizes at the top of the list. Such elements are able to picked out in mpralm due to its information sharing and weighting framework.

**Fig. 8:**
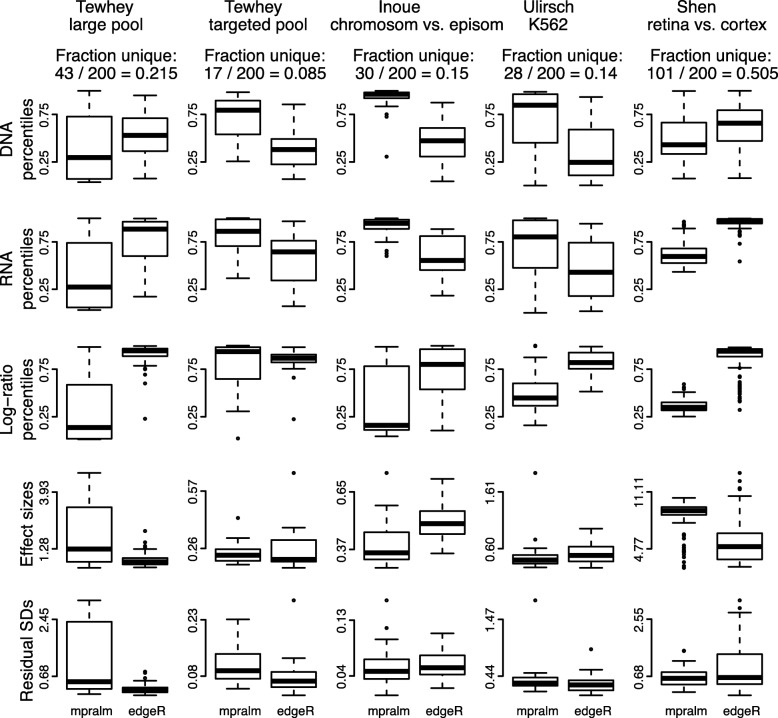
Distribution of quantities related to statistical inference in top ranked elements with mpralm and edgeR. Similar to Fig. 7

**Fig. 9:**
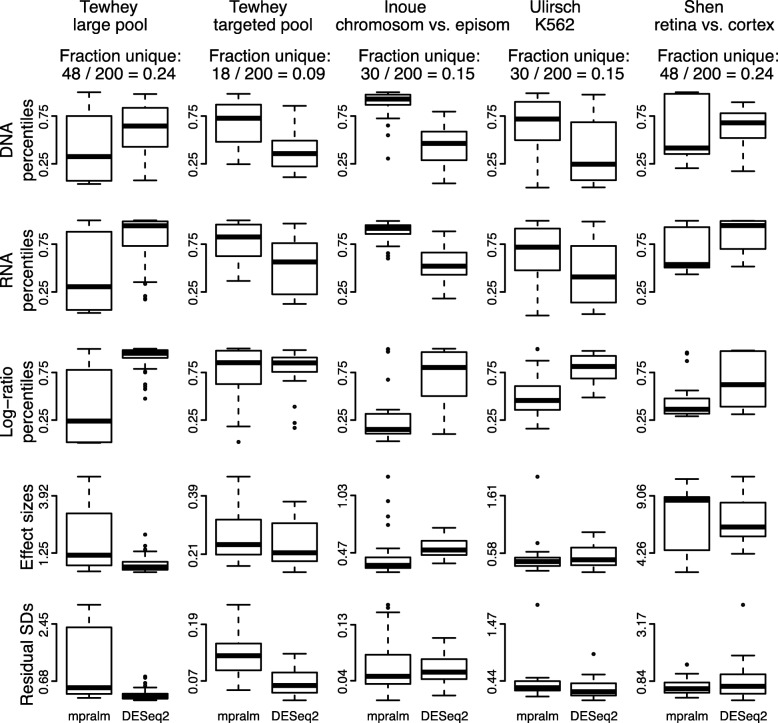
Distribution of quantities related to statistical inference in top ranked elements with mpralm and DESeq2. Similar to Fig. 7

We similarly compare mpralm rankings with edgeR and DESeq2 rankings in Figs. 8 and 9. The ranking concordance between mpralm and these two methods is much higher than with the t-test. Generally, uniquely top ranking mpralm elements have higher DNA and RNA levels, but lower log-ratio activity measures. Uniquely top ranking mpralm elements also tend to have larger effect sizes. The variability of activity measures (residual SD) is similar among the methods.

### Accuracy of activity measures and power of differential analysis depends on summarization technique over barcodes

MPRA data initially contain count information at the barcode level, but we typically desire information summarized at the element level for the analysis stage. We examine the theoretical properties of two summarization methods: averaging and aggregation. Under the assumption that DNA and RNA counts follow a count distribution with a mean-variance relationship, we first show that averaging results in activity estimates with more bias. Second, we examine real data performance of these summarization techniques.

Let *R*_*b*_ and *D*_*b*_ denote the RNA and DNA count, respectively, for barcode *b*=1,…,*B* for a putative regulatory element in a given sample. We suppress the dependency of these counts on sample and element. Typically, *B* is approximately 10 to 15 (for examples, see Table 1). We assume that *R*_*b*_ has mean *μ*_*r*_ and variance *k*_*r*_*μ*_*r*_ and that *D*_*b*_ has mean *μ*_*d*_ and variance *k*_*d*_*μ*_*d*_. Typically the constants *k*_*d*_ and *k*_*r*_ are greater than 1, modeling overdispersion. Negative binomial models are a particular case with *k*=1+*ϕμ*, where *ϕ* is an overdispersion parameter. Also let *N*_*d*_ and *N*_*r*_ indicate the library size for DNA and RNA, respectively, in a given sample. Let *p*_*d*_ and *p*_*r*_ indicate the fraction of reads mapping to element *e* for DNA and RNA, respectively, in a given sample so that *μ*_*r*_=*N*_*r*_*p*_*r*_ and *μ*_*d*_=*N*_*d*_*p*_*d*_. Let *a* be the true activity measure for element *e* defined as *a*:= log(*p*_*r*_/*p*_*d*_). When performing total count normalization, the RNA and DNA counts are typically scaled to a common library size *L*.

The average estimator of *a* is an average of barcode-specific log activity measures:

$$\hat a^{AV} = \frac{1}{B} \sum\limits_{b = 1}^{B} \log\left(\frac{R_{b}L/N_{r} + 1}{D_{b}L/N_{d} + 1} \right) $$

Using a second order Taylor expansion (Methods), it can be shown that this estimator has bias approximately equal to

$$\text{bias}^{AV} \approx \frac{1}{2}\left(\frac{k_{d}}{\mu_{d}} - \frac{k_{r}}{\mu_{r}} \right) = \frac{1}{2}\left(\frac{k_{d}}{N_{d} p_{d}} - \frac{k_{r}}{N_{r} p_{r}} \right) $$

The aggregate estimator of *a* first aggregates counts over barcodes:

$$\hat a^{AGG} = \log\left(\frac{1 + (L/N_r)\sum\nolimits_{b=1}^{B} R_{b}}{1 + (L/N_d)\sum\nolimits_{b=1}^{B} D_{b}} \right) $$

Using an analogous Taylor series argument (Methods), we can show that this estimator has bias approximately equal to

$$\text{bias}^{AGG} \approx \frac{1}{B}\text{bias}^{AV} $$

The aggregate estimator has considerably less bias than the average estimator for most MPRA experiments because most experiments use at least 10 barcodes per element. Bias magnitude depends on count levels and the true activity measure *a*. Further, the direction of bias depends on the relative variability of RNA and DNA counts. Similar Taylor series arguments show that the variance of the two estimators is approximately the same.

**Fig. 10:**
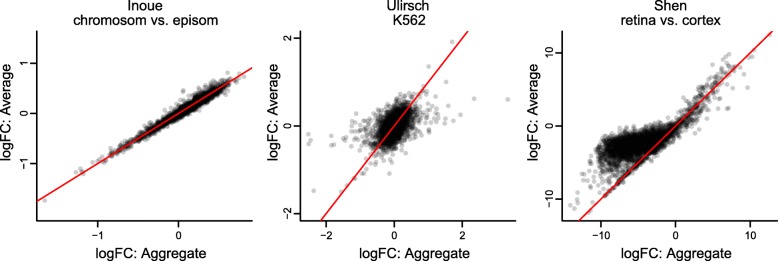
Comparison of the average and aggregate estimators For the three datasets containing barcode-level information, we compare the effect sizes (log fold changes in activity levels) resulting from use of the aggregate and average estimators. The *y*=*x* line is shown in red

The choice of estimator can impact the estimated log fold-changes (changes in activity) in a differential analysis. In Fig. 10 we compare the log fold-changes inferred using the two different estimators. For the Inoue dataset, these effect sizes are very similar, but there are larger differences for the Ulirsch and Shen datasets.

Aggregation technique affects power in a differential analysis. In the last three columns of Figs. 3, 4, 5, and 6, we compare aggregation to averaging using mpralm. The two estimators have similar type I error rates but very different detection rates between datasets. The average estimator is more favorable for the Ulirsch and Shen datasets, and the aggregate estimator is more favorable in the Inoue dataset.

### Recommendations for sequencing depth and sample size

**Fig. 11:**
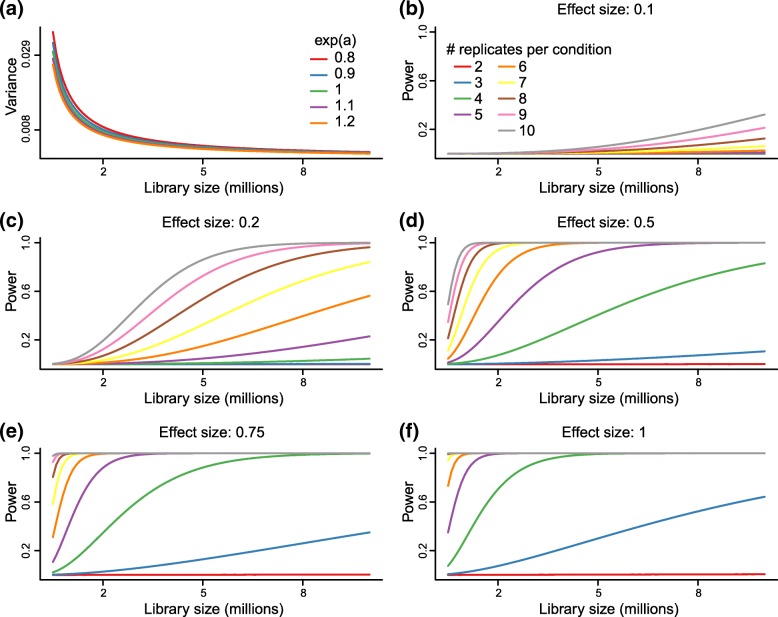
Power analysis. Variance and power calculated based on our theoretical model. **(a)** Variance of the aggregate estimator depends on library size and the true unknown activity level but not considerably on the latter. **(b)**-**(f)** Power curves as a function of library size for different effect sizes and sample sizes. Effect sizes are log2 fold-changes

To aid in the design of future MPRA experiments, we used the above mathematical model to inform power calculations. Power curves are displayed in Fig. 11. We observe that the variance of the aggregate estimator depends minimally on the true unknown activity measure but is greatly impacted by sequencing depth. We fix one of the two true activity measures to be 0.8 as this is common in many datasets. We use a nominal type I error rate of 0.05 that has been Bonferroni adjusted for 5000 tests to obtain conservative power estimates. We also use ten barcodes per element as this is typical of many studies.

**Fig. 12:**
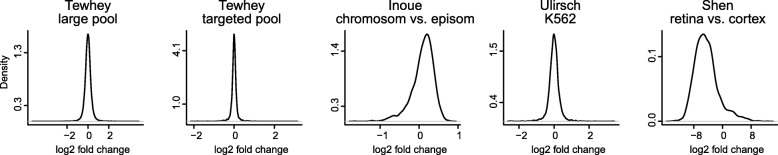
Effect size distributions across datasets. Effect sizes in MPRA differential analysis are the (precision-weighted) differences in activity scores between groups, also called log2 fold-changes. The distribution of log2 fold changes resulting from using mpralm with the aggregate estimator are shown here

Our model suggests different impacts of sample size, and a marked impact of increasing the number of replicates, especially between 2 and 6 samples. From Fig. 12, we can see that large effect sizes (effect sizes of 1 or greater) are typical for top ranking elements in many MPRA studies. In this situation it is advisable to do 4 or more replicates per group.

## Discussion

The field of MPRA data analysis has been fragmented and consists of a large collection of study-specific ad hoc methods. Our objective in this work has been to provide a unified framework for the analysis of MPRA data. Our contributions can be divided into three areas. First, we have investigated techniques for summarizing information over barcodes. In the literature, these choices have always been made without justification and have varied considerably between studies. Second, we have developed a linear model framework, mpralm, for powerful and flexible differential analysis. To our knowledge, this is the second manuscript evaluating for statistical analysis in MPRA studies. The first proposed the QuASAR-MPRA method [23], which we show to have worse performance than mpralm. In our comparisons, we provide the largest and most comprehensive comparison of analysis methods so far; earlier work used only two datasets for comparisons. Third, we have analyzed the impact of sequencing depth and number of replicates on power. To our knowledge, this is the first mathematically-based power investigation, and we expect this information to be useful in the design of MPRA studies.

The activity of a regulatory element can be quantified with the log-ratio of RNA counts to DNA counts. In the literature, groups have generally taken two approaches to summarizing barcode information to obtain one such activity measure per element per sample. One approach is to add RNA and DNA counts from all barcodes to effectively increase sequencing depth for an element. This is termed the aggregate estimator. Another approach is to compute the log-ratio measure for each barcode and use an average of these measures as the activity score for an element. This is termed the average estimator, and we have shown that it is more biased than the aggregate estimator. Because of this bias, we caution against the use of the average estimator when comparing activity scores in enhancer groups (often defined by sequence features). However, it is unclear which of the two estimators is more appropriate for differential analysis.

In addition to barcode summarization recommendations, we have proposed a linear model framework, mpralm, for the differential analysis of MPRA data. Our evaluations show that it produces calibrated *p*-values and is as or more powerful than existing methods being used in the literature.

While the count-based tools, edgeR and DESeq2, would seem like natural methods to use for the analysis of MPRA data, they have not yet been used for differential analysis of MPRA activity measures between groups. There has been some use of DESeq2 to identify (filter) elements with regulatory activity (differential expression of RNA relative to DNA) [19, 32]. However, these tools have not been used for comparisons of activity measures between groups. In this work we propose the use of log-DNA offsets as potential sensible uses of these software for differential analysis. In our evaluations, we see that this approach is most competitive with mpralm. For the allelic studies [18, 19], we observe that the degree of within-sample correlation affects the power of mpralm relative to comparison methods. In particular, there is little difference in the performance of the different methods for the Tewhey large pool experiment, and this experiment had overall low within-sample correlation. Both the Tewhey targeted pool experiment and the Ulirsch experiment had larger within-sample correlations, and we observe that mpralm has increased power over the comparison methods for these datasets. We expect that mpralm will generally be more powerful for paired designs with high within-pair correlations.

In terms of element rankings, mpralm, edgeR, and DESeq2 are similar. However, we observe a substantial difference in ranking between t-tests and mpralm and believe top ranked mpralm elements exhibit better properties compared to those from t-tests.

Linear models come with analytic flexibility that is necessary to handle diverse MPRA designs. Paired designs involving alleles, for example, are easily handled with linear mixed effects models due to computational tractability. The studies we have analyzed here only consider two alleles per locus. It is possible to have more than two alleles at a locus, and such a situation cannot be addressed with paired t-tests, but is easily analyzed using mpralm. This is important because we believe such studies will eventually become routine for understanding results from genome-wide association studies. We note that for allelic studies, it is often of interest to filter detections of significant differences to those cases where at least one allele appears to show regulatory activity. This is not inherent in the mpralm method, but it is possible to screen for regulatory activity with a conventional count-based differential analysis of RNA counts versus DNA counts using methods such as voom, edgeR, or DESeq2.

While we have focused on characterizing the mpralm linear model framework for differential analysis, it is possible to include variance weights in the multivariate models used in saturation mutagenesis and characterization studies. We expect that modeling the copy number-variance relationship will improve the performance of these models.

For power, we find a substantial impact of even small increases in sample size. This is an important observation because many MPRA studies use 2 or 3 replicates per group, and our results suggest that power can be substantially increased with even a modest increase in sample size. We caution that using less than 4 replicates can be quite underpowered.

In short, the tools and ideas set forth here will aid in making rigorous conclusions from a large variety of future MPRA studies.

## Conclusions

We have observed differences in the MPRA activity estimates resulting from the averaging and aggregation methods of summarizing counts across barcodes. For this reason, we recommend that practitioners perform sensitivity analyses for their results by using both of these estimation procedures. The mpralm linear model framework appears to have calibrated type I error rates and to be as or more powerful than the t-tests and Fisher’s exact type tests that have been primarily used in the literature. mpralm has similar performance to variations of the edgeR and DESeq2 methods that we introduce here. These variations involve including DNA counts as offsets in the RNA differential analysis procedures. We recommend either of these 3 methods for unpaired differential analysis settings (such as tissue comparison studies), but we recommend mpralm for allelic studies due to its ability to better model the paired nature of the alleles with mixed models. Finally, we recommend that practitioners use at least 4 samples per condition for reasonable power to detect differences for top ranking elements.

## Methods

### Data

See Table 1. Dataset labels used in figures are accompanied by short descriptions below.

**Melnikov**: Study of the base-level impact of mutations in two inducible enhancers in humans [12]: a synthetic cAMP-regulated enhancer (CRE) and a virus-inducible interferon-beta enhancer (IFNB). We do not look at the IFNB data because it contains only one sample. We consider 3 datasets:

**Melnikov: CRE, single-hit, induced state**: Synthetic cAMP-regulated enhancer, single-hit scanning, induced state.

**Melnikov: CRE, multi-hit, uninduced state**: Synthetic cAMP-regulated enhancer, multi-hit sampling, uninduced state.

**Melnikov: CRE, multi-hit, induced state**: Synthetic cAMP-regulated enhancer, multi-hit sampling, induced state.

**Kheradpour**: Study of the base-level impact of mutations in various motifs [15]. Transfection into HepG2 and K562 cells.

**Tewhey**: Study of allelic effects in eQTLs [19]. Transfection into two lymphoblastoid cell lines (NA12878 and NA19239) as well as HepG2. In addition two pools of plasmids are considered: a large screening pool and a smaller, targeted pool, designed based on the results of the large pool. We use data from both the large and the targeted pool in NA12878.

**Inoue: chromosomal vs. episomal**: Comparison of episomal and chromosomally-integrated constructs [21]. This study uses a wild-type and mutant integrase to study the activity of a fixed set of putative regulatory elements in an episomal and a chromosomally-integrated setting, respectively.

**Ulirsch**: Study of allelic effects in GWAS to understand red blood cell traits [18]. Transfection into K562 cells as well as K562 with GATA1 overexpressed. We use the data from K562.

**Shen: mouse retina vs. cortex**: Comparison of cis-regulatory elements in-vivo in mouse retina and cerebral cortex [22]. Candidate CREs that tile targeted regions are assayed in-vivo in these two mouse tissues with adeno-associated virus delivery.

### Simulation of data for type I error rate estimation

Here we describe the realistic simulation of data to closely match properties of the real datasets. The starting point is a given dataset with two comparison groups:

1. Compute log-ratio activity measures from the original RNA and DNA counts.
2. Calculate and save the element-wise residual standard deviations of the log-ratios after mean-centering them in each group. Calculate and save the mean of the original log-ratios in group 1. These element-wise means will become the mean for both groups in the new null data.
3. Standardize the log-ratios in each group to have mean zero and unit variance.
4. The standardized log-ratios from both groups all have mean zero and unit variance. Resample these standardized residuals without replacement for each element in each sample. For paired (allelic) studies, resample each allele-allele pair without replacement.
5. Multiply the resampled residuals by the element-wise residual standard deviations, and add the original group 1 element-specific means. This creates identically distributed log-ratios in both comparison groups.
6. Retain the original DNA counts from all samples.
7. Compute RNA counts using the original DNA counts and the resampled log-ratio activity measures.
8. The original DNA counts and the new RNA counts form the new synthetic dataset.

### Count preprocessing

DNA and RNA counts are scaled to have the same library size before running any methods. We perform minimal filtering on the counts to remove elements from the analysis that have low counts across all samples. Specifically, we require that DNA counts must be at least 10 in all samples to avoid instability of the log-ratio activity measures. We also remove elements in which these log-ratios are identical across all samples; in practice this only happens when the RNA counts are zero across all samples. Both filtering steps remove clear outliers in the copy number-variance plot (Fig. 2).

### Modeling

The square root of the standard deviation of the log-ratios over samples is taken as a function of average log DNA levels over samples, and this relationship is fit with a lowess curve. Predicted variances are inverted to form observation-level precision weights. Log-ratios activity measures and weights are used in the voom analysis pipeline. For the allelic studies, a mixed model is fit for each element using the duplicateCorrelation module in the limma Bioconductor package [33].

This linear model approach has a number of advantages. (1) It is flexible to different functional forms of the variance-copy number relationship. (2) It allows for a unified approach to modeling many different types of MPRA design using the power of design matrices. (3) It allows for borrowing of information across elements using empirical Bayes techniques. (4) It allows for different levels of correlation between elements using random effects.

### mpralm enables modeling for complex comparisons

While many comparisons of interest in MPRA studies can be posed as a two group comparison (e.g. major allele vs. minor allele), more complicated experimental designs are also of interest. For example, in the allelic study conducted by Ulirsch [18], putative biallelic enhancer sequences are compared in two cellular contexts. The first is a standard culture of K562 cells, and the second is a K562 culture that induces over-expression of GATA1 for a more terminally-differentiated phenotype. A straightforward question is whether an allele’s effect on enhancer activity differs between cellular contexts. Let *y*_*eia*_ be the enhancer activity measure (log ratio of RNA over DNA counts) for element *e*, in sample *i* for allele *a*. Let *x*_1*eia*_ be a binary indicator of the mutant allele. Let *x*_2*eia*_ be a binary indicator of the GATA1 over-expression condition. Then the following model

$$\begin{array}{*{20}l} Y_{eia} =& \beta_{0e} + \beta_{1e}x_{1eia} + \beta_{2e}x_{2eia} + \\ & \beta_{3e}x_{1eia}x_{2eia} + b_{i} + \epsilon_{eia} \end{array} $$

is a linear mixed effects model for activity measures, where *b*_*i*_ is a random effect that induces correlation between the two alleles measured within the same sample. We can perform inference on the *β*_3*e*_ parameters to determine differential allelic effects. Such a model is easy to fit within the mpralm framework, since our framework supports model specifications by general design matrices. In contrast, this question cannot be formulated in the QuASAR, t-test, and Fisher’s exact test frameworks. Neither edgeR nor DESeq2 support the fitting of mixed effects models.

### Running mpralm, QuASAR, t-test, fisher’s exact test

For all methods, DNA and RNA counts were first corrected for library size with total count normalization. For edgeR and DESeq2, DNA counts were included as offset terms on the log scale before standard analysis. For the t-test we computed the aggregate estimator of the log-ratio as the outcome measure. For Fisher’s exact test, we summed DNA and RNA counts in the two conditions to form a 2-by-2 table as input to the procedure. For QuASAR-MPRA, we summed RNA counts in each condition to get one reference condition count and one alternative condition count per element. We also summed DNA counts in all samples and in the reference condition to get one DNA proportion for each element. These were direct inputs to the method.

### Metrics used for method comparison

We use a number of metrics to compare methods and describe them in detail here.

- **Shape of*p*-value distributions.** Calibrated differential analysis methods have a characteristic shape for the *p*-value distribution. Normally the majority of *p*-values are uniformly distributed, corresponding to null comparisons, and there is a peak at low *p*-values for the truly differential comparisons. We compare the methods with regards to this expected shape.
- **Type I error rates.** For all methods and datasets, we estimate via realistic simulation (described above) the proportion of truly null comparisons in which we reject the null hypothesis.
- **Number of rejections as a function of type I error rate.** To some degree, this is a comparison of power between methods. We cannot compare power of methods by comparing the height of the peaks around zero in the *p*-value distributions because those plots tell us nothing about type I error rates. We wished to fix type I error rate and compare the number of rejections made by the different methods. For a given *nominal* type I error rate, we computed (1) the number of rejections made by a method and (2) the estimated true type I error rate. For example, a conservative method would have a true type I error rate of 0.03, say, at a nominal level of 0.05. Quantity (2) is plotted on the x-axis of Fig. 5, and quantity (1) is plotted on the y axis. Curves that are above others indicate higher detections for fixed type I error rate.
- **False discovery rates.** These are defined as the fraction of rejections that are false positives. The estimation of these FDRs is described below.
- **Metrics that describe top ranking elements.** The metrics above focus on type I error rates and power. Comparisons of the element rankings produced by the different methods were performed by taking elements that were ranked in the top 200 for one method but not the other. (Comparisons were done pairwise with mpralm always being one method compared.) Metrics measured the magnitude of the RNA counts, DNA counts, estimated log-ratio activity measures, effect sizes (difference in activity between groups), and residual standard deviations of the activity measures. It is nice if the RNA, DNA, log-ratios, and effect sizes are higher in top ranking elements. It is nice if the top ranking elements have residual standard deviations that are low, but not so low as to have been underestimated. It is common for variability to be underestimated when there are uniformly low counts across samples.

### Estimation of FDR

The proportion of truly null hypotheses *π*_0_ for each dataset was estimated using the “lfdr” method in the propTrueNull function within limma [31]. As is common with *π*_0_ estimation procedures, the *p*-values resulting from a statistical analysis are used in the estimation process. To this end, the *π*_0_ proportion was estimated with the *p*-values resulting from mpralm, t-test, QuASAR, edgeR, and DESeq2, and the median of these estimates was used as the estimate for *π*_0_ for a given dataset. Fisher’s exact test was excluded from this estimate because it gave an estimate of *π*_0_ that was considerably smaller than the other methods, and which was dubious in light of its uncontrolled type I error rate. We multiply the number of tests by these *π*_0_ estimates to obtain an estimate of the number of truly null hypotheses. We then multiply this by our estimate of the true dataset-specific type I error rate (as shown in Fig. 4) to obtain an estimate of the number of false positives. Dividing by the number of rejected hypotheses at a given nominal significance level gives the estimated FDRs in Fig. 6.

### Bias and variance of estimators

We use Taylor series arguments to approximate the bias and variance of the aggregate and average estimators. The following summarizes our parametric assumptions:

$$\begin{array}{*{20}l} \mathrm{E}[R_{b}] &= \mu_{r} = N_{r} p_{r} & \text{Var}(R_{b}) &= k_{r}\mu_{r} \\ \mathrm{E}[D_{b}] &= \mu_{d} = N_{d} p_{d} & \text{Var}(D_{b}) &= k_{d}\mu_{d} \end{array} $$

We suppress the dependency of these parameters on sample and element. Library sizes are given by *N*. The fraction of reads coming from a given element is given by *p*. Dispersion parameters are given by *k*. The common library size resulting from total count normalization is given by *L*. The true activity measure of a given element is given by *a*:= log(*p*_*r*_/*p*_*d*_).

**Average estimator:** The “average estimator” of *a* is an average of barcode-specific log activity measures and is written as:

$$\hat a^{AV} = \frac{1}{B} \sum\limits_{b = 1}^{B} \log\left(\frac{R_{b}L/N_{r} + 1}{D_{b}L/N_{d} + 1} \right) $$

The second-order Taylor expansion of the function

$$f(R_{b},D_b) = \log(R_{b} L/N_{r} + 1) - \log(D_{b} L/N_{d} + 1) $$

around the point (E[*R*_*b*_],E[*D*_*b*_])=(*μ*_*r*_,*μ*_*d*_) is:

$$\begin{aligned} \log& \left(\frac{R_{b} L/N_{r} +1}{D_{b} L/N_{d} +1} \right) \approx \\ & \log\left(\mu_{r} L/N_{r} +1 \right) - \log\left(\mu_{d} L/N_{d} +1 \right) \\ &+ \frac{L/N_{r}}{\mu_{r}L/N_{r}+1}(R_{b} - \mu_{r}) \\ &- \frac{L/N_{d}}{\mu_{d}L/N_{d}+1}(D_{b} - \mu_{d}) \\ &- \frac{(L/N_{r})^{2}}{2(\mu_{r}L/N_{r}+1)^{2}}(R_{b} - \mu_{r})^{2} \\ &+ \frac{(L/N_{d})^{2}}{2(\mu_{d}L/N_{d}+1)^{2}}(D_{b}-\mu_{d})^{2} \end{aligned} $$

We use the expansion above to approximate the expectation of the average estimator:

$$\begin{array}{*{20}l} \mathrm{E}\left[ \hat a^{AV} \right] & \approx \log\left(\frac{\mu_{r}L/N_{r}+1}{\mu_{d}L/N_{d}+1} \right) \\ &\quad + \frac{(L/N_{d})^{2} k_{d}\mu_{d}}{2(\mu_{d}L/N_{d}+1)^{2}} - \frac{(L/N_{r})^{2} k_{r}\mu_{r}}{2(\mu_{r}L/N_{r}+1)^{2}} \\ &\approx \log\left(\frac{p_{r}}{p_{d}} \right) + \frac{k_{d}}{2\mu_{d}} - \frac{k_{r}}{2\mu_{r}} \\ &= a + \frac{k_{d}}{2\mu_{d}} - \frac{k_{r}}{2\mu_{r}} \end{array} $$

We can also approximate the variance under the assumption that the barcode-specific log-ratios are uncorrelated:

$$\begin{array}{*{20}l} \text{Var}(\hat a^{AV}) &= \frac{1}{B} \text{Var} \left(\log\left(\frac{R_{b}L/N_{r}+1}{D_{b}L/N_{d}+1} \right) \right) \\ &\approx \frac{(L/N_{r})^{2} k_{r}\mu_{r}}{B(\mu_{r} L/N_{r} + 1)^{2}} + \frac{(L/N_{d})^{2} k_{d}\mu_{d}}{B(\mu_{d} L/N_{d} + 1)^{2}} \\ &\quad - \frac{2 (L/N_{r}) (L/N_{d}) \text{Cov}(R_{b}, D_{b})}{B(\mu_{r} L/N_{r} + 1)(\mu_{d} L/N_{d} + 1)} \end{array} $$

**Aggregate estimator:** The “aggregate estimator” of *a* first aggregates counts over barcodes and is written as:

$$\begin{array}{*{20}l} \hat{a}^{AGG} &= \log\left(\frac{1 + (L/N_{r})\sum\nolimits_{b=1}^{B} R_{b}}{1 + (L/N_{d})\sum\nolimits_{b=1}^{B} D_{b}} \right) \\ &= \log \left(\frac{1 + (L/N_{r})R^{AGG}}{1 + (L/N_{d})D^{AGG}} \right) \end{array} $$

The second-order Taylor expansion of the function

$$ {\begin{aligned} f(R^{AGG},D^{AGG}) = \log((L/N_{r})R^{AGG}+1) - \log((L/N_{d})D^{AGG}+1) \end{aligned}} $$

around the point (E[*R*^*AGG*^],E[*D*^*AGG*^])=(*Bμ*_*r*_,*Bμ*_*d*_) is:

$$\begin{aligned} \log & \left(\frac{1 + (L/N_{r})R^{AGG}}{1 + (L/N_{d})D^{AGG}} \right) \approx \\ & \log\left(B\mu_{r}L/N_{r} +1 \right) - \log\left(B\mu_{d}L/N_{d} +1 \right) \\ & + \frac{L/N_{r}}{B\mu_{r}L/N_{r} +1}(R^{AGG} - B\mu_{r}) \\ & - \frac{L/N_{d}}{B\mu_{d}L/N_{d} +1}(D^{AGG} - B\mu_{d}) \\ & - \frac{(L/N_{r})^{2}}{2(B\mu_{r}L/N_{r} +1)^{2}}(R^{AGG}- B\mu_{r})^{2} \\ & + \frac{(L/N_{d})^{2}}{2(B\mu_{d}L/N_{d} +1)^{2}}(D^{AGG}-B\mu_{d})^{2} \end{aligned} $$

We use the expansion above to approximate the expectation:

$$\begin{array}{*{20}l} \mathrm{E}\left[ \hat a^{AGG} \right] &\approx \log\left(\frac{B\mu_{r}L/N_{r}+1}{B\mu_{d}L/N_{d}+1} \right) \\ &\quad + \frac{B k_{d}\mu_{d} (L/N_{d})^{2}}{2(B\mu_{d}L/N_{d} +1)^{2}} \\ &\quad - \frac{B k_{r}\mu_{r} (L/N_{r})^{2}}{2(B\mu_{r}L/N_{r} +1)^{2}} \\ &\approx \log\left(\frac{p_{r}}{p_{d}} \right) + \frac{k_{d}}{2B\mu_{d}} - \frac{k_{r}}{2B\mu_{r}} \\ &= a + \frac{k_{d}}{2B\mu_{d}} - \frac{k_{r}}{2B\mu_{r}} \end{array} $$

We can also approximate the variance:

$$\begin{array}{*{20}l} \text{Var} & (\hat a^{AGG}) \approx \\ &\frac{(L/N_{r})^{2} B k_{r}\mu_{r}}{(B\mu_{r} L/N_{r} + 1)^{2}} + \frac{(L/N_{d})^{2} B k_{d}\mu_{d}}{(B\mu_{d} L/N_{d} + 1)^{2}} \\ &- \frac{2 (L/N_{r}) (L/N_{d}) \text{Cov}(R^{AGG}, D^{AGG})}{(B\mu_{r} L/N_{r} + 1)(B\mu_{d} L/N_{d} + 1)} \end{array} $$

## Abbreviations

FET: Fisher’s exact test
LCL: Lymphoblastoid cell line
MPRA: Massively parallel reporter assay
